# A feasibility study to determine the use of baited pots in Greenland halibut (*Reinhardtius hippoglossoides*) fisheries, supported by the use of underwater video observations

**DOI:** 10.7717/peerj.10536

**Published:** 2021-01-04

**Authors:** Margaret H. Folkins, Scott M. Grant, Philip Walsh

**Affiliations:** Center for Sustainable Aquatic Resources, Memorial University of Newfoundland, St. John’s, Newfoundland and Labrador, Canada

**Keywords:** Greenland halibut, Underwater video, Fishing gear, Behaviour, Nunavut, Sustainability, Conservation, Fisheries

## Abstract

High incidental catches of Greenland shark (*Somniosus microcephalus*) in Nunavut’s Greenland halibut (*Reinhardtius hippoglossoides*) fishery has led to studies on the feasibility of capturing Greenland halibut with baited pots. In this study, catch rates among six experimental pots are compared. In addition to this, underwater video observations of Greenland halibut interacting with two of these experimental pot types are quantified in order to help provide recommendations on future pot designs. Catch rates of Greenland halibut differed among pots with different entrance mesh types, and none of the pots produced substantial amounts of bycatch. Strings of pots were deployed within a narrow corridor between baited gillnets targeting Greenland halibut, which may have affected catch results. Video observations revealed Greenland halibut entangled by their teeth significantly more often in entrance funnels constructed with 50 mm than with 19 mm clear monofilament netting and the entrance rate was 45% higher with the 19 mm netting. Greenland halibut that successfully entered a pot repeatedly became entangled by their teeth in 58 mm netting used in the side and end panels and in a horizontal panel used to separate the pot into a lower and upper chamber. The majority (80%) of Greenland halibut were observed to approach a pot against the current. The downstream entrance was aligned with the current in 52% of the observed Greenland halibut approaches. Seventy percent of entry attempts and 67% of successful entries occurred when fish approached against the current and when the entrance was aligned with the current. These observations lead to recommendations that future studies consider developing a four entrance pot to ensure an entrance is always aligned with bottom currents. Based on these observations of entanglements, it is recommended to use 19 mm clear monofilament netting in the entrance funnel, 100 mm polyethylene netting in the exterior panels, and 19 mm polypropylene netting in the horizontal panel when targeting Greenland halibut. Three Greenland sharks were observed interacting with the pots in the video sets, but none were captured or damaged the pots during the potting experiments, providing validity to the use of pots to mitigate the capture of Greenland shark in Nunavut territorial waters.

## Introduction

The Greenland halibut (*Reinhardtius hippoglossoides*) fishery is the most profitable groundfish fishery in the inshore territorial waters of Nunavut, Canada (i.e., Nunavut Settlement Area; NSA) and adjacent offshore waters within Baffin Bay and Davis Strait (i.e., NAFO Divisions 0A and 0B) (Fisheries and Oceans Canada, DFO)(2014). The NSA includes waters directly adjacent to Nunavut, extending 12 miles from land within Canada’s territorial zone.

In the Northwest Atlantic, including NAFO Divisions 0A and 0B, gillnets and longlines are the fixed gears used to capture Greenland halibut (halibut, hereafter) (Fisheries and Oceans Canada, DFO)(2014). Longlines are used in the NSA while gillnets, and to a lesser extent longlines, are used in adjacent offshore waters. Nunavut communities are highly dependent on their marine resources for survival and economic prosperity (DFO, 2006; Treble & Stewart, 2009). However, pressures from fishing industries can cause adverse impacts to the marine environment that can have negative impacts on ecosystems and threaten the long-term sustainability of fishery resources (Innes & Pascoe, 2010). Areas of concern include the capture of non-targeted species and destruction of seabed habitat (Cashion et al., 2018; Fangel et al., 2015). Research on methods to mitigate these impacts is of value from both industry and environmental perspectives (Fuller et al., 2008; NOAA, 2017).

Although longline and gillnet fisheries are efficient at capturing halibut, non-targeted species, including many species of invertebrates, fish, seabirds, and cetaceans are also vulnerable to capture (Fangel et al., 2015; He, 2005; Walsh, 2008; Young, 2010). For example, as a result of concerns regarding potential bycatch of large marine mammals and Greenland shark (*Somniosus microcephalus*), gillnets were banned in the halibut fishery in Cumberland Sound, Nunavut (DFO, 2014). Moreover, high bycatch rates of Greenland shark in exploratory halibut longline fisheries within the NSA (Walsh, 2008; Young, 2010) have led to research into modifying longline gear to avoid its capture (Grant, Sullivan & Hedges, 2018; Grant, 2015). In addition to capture of non-target species, long soak times in the halibut offshore gillnet fishery (≥5 days) can often lead to the capture of fish of lower quality, including partially eaten or decomposed fish, likely resulting in lower profits to industry (Savina et al., 2016).

The introduction of baited pots in halibut fixed gear fisheries within the NSA and adjacent offshore waters has the potential to provide substantial environmental benefits as well as economic gains to the fishing industry. For example, many of the non-targeted species captured in pots can be released alive and unharmed (Grant, 2015). In the event of inclement weather, soak time has limited influence on market quality of pot-caught fish, and ghost fishing of lost pots can be prevented with the use of biodegradable materials (Suuronen et al., 2012; Thomsen, Humborstad & Furevik, 2010). Discarding of targeted species captured in gillnet and longline fisheries, resulting from death, decomposition, and damage from scavengers, is reduced in potting fisheries and pots can be modified to avoid harvesting of undersized fish by adjusting mesh size (Hedgärde et al., 2016; Königson et al., 2015a; Königson et al., 2015b; Ovegård et al., 2011). By introducing pots, loss of product from Greenland shark or cetacean depredation (Pike, 1994; Dyb, 2006; Mesnick et al., 2006) can be eliminated and costs can be reduced as less bait is needed and tending baited pots is less labour-intensive than methods currently used in Nunavut’s halibut fixed gear fisheries (Grant, 2015).

Initial efforts to use baited pots to capture halibut in Canadian waters were carried out using Newfoundland-style cod pots (Newfoundland pot, hereafter) that as a result of their large size, are fished individually (Murphy, 2014). The pots used by Murphy (2014) failed to capture appreciable quantities of halibut in Newfoundland waters. This failure may have been related to the fact that Atlantic cod (*Gadus morhua*) and halibut differ considerably in morphology and behaviour and may therefore require different pot designs and potting strategies to successfully capture them. More recent potting studies have been carried out with Norwegian-style cod pots (Norwegian pot, hereafter) (Grant, 2015) which are smaller, less expensive, light weight, and can be fished in strings of several pots. Fishing several smaller pots in a string allows the halibut fishing industry to cover a larger area of the seabed, similar to longlines and gillnets. Results from the Norwegian pots were encouraging, with up to 20 halibut (32 kg) captured in a pot in overnight sets (Grant, 2015). Moreover, pots were found to outperform longlines, capturing five times more halibut when catches were standardized for the linear distance of the fishing gears on the seabed (Grant, 2015). With regard to halibut, a preliminary study involving underwater video camera observations revealed that halibut became entangled by their teeth in the monofilament diamond netting of the entrance funnel and square mesh netting in the side panels of a Norwegian pot (Grant, 2015).

Globally, fishing gear technologists are struggling to find ways to maximize catch rates in order to make pots more commercially appealing (Eayrs & Pol, 2019; Suuronen et al., 2012). When fishing gear studies are based on catch rate data alone, they suffer from a lack of knowledge with regard to capture efficiency. For example, if 100 fish approach a baited pot but only 10 are landed, then the capture efficiency is only 10%. By observing the number of fish that approach a baited pot and the behaviour of these fish as they interact with a pot, we can assess the effects of varying the design features and construction materials so as to maximize the number of fish that enter a pot and minimize the number escaping (Anders et al., 2017; Meintzer, Walsh & Favaro, 2018; Winger, Løkkeborg & Pol, 2016).

**Figure 1:**
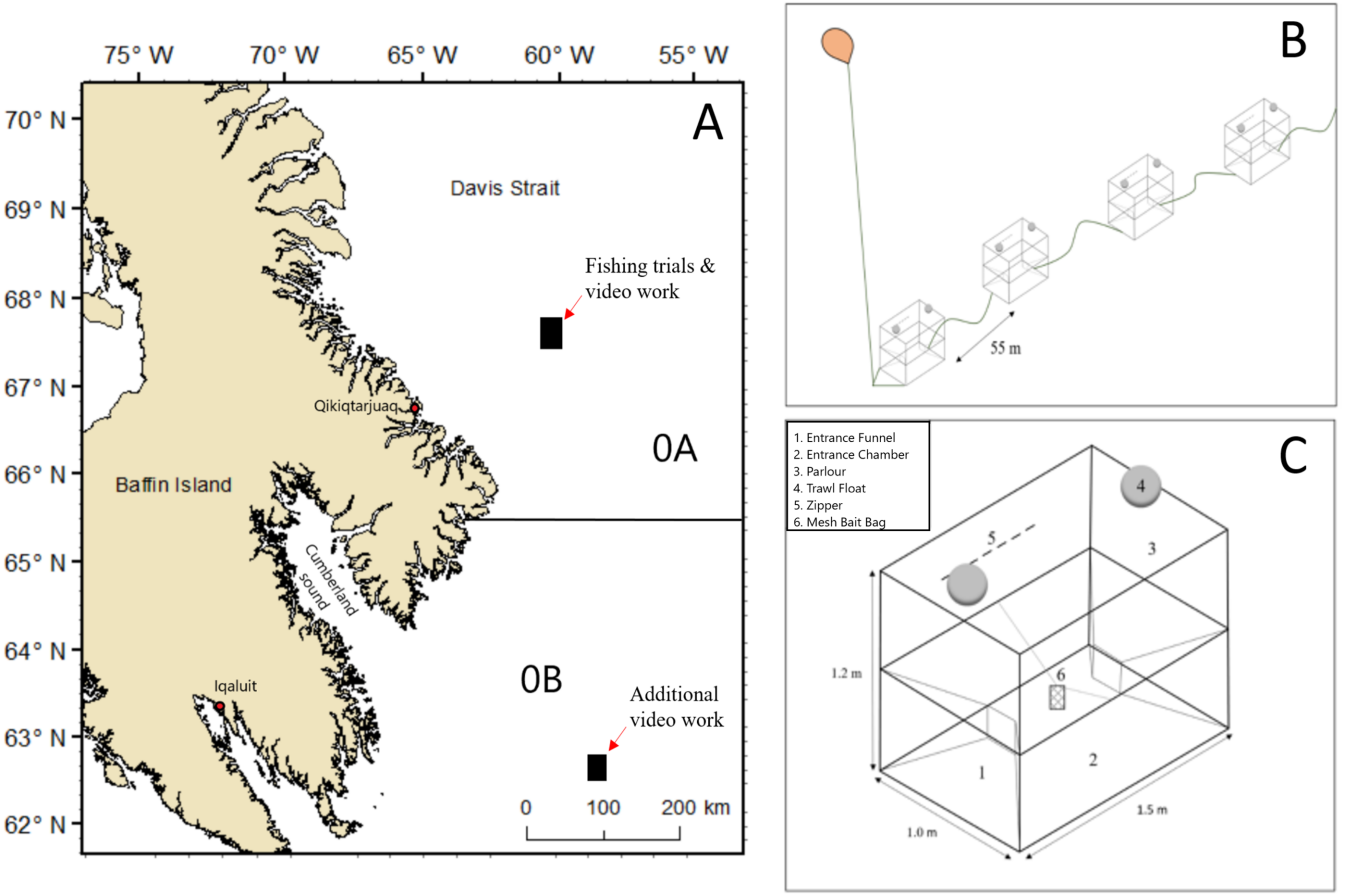
Map showing areas in NAFO Division 0A and 0B where fishing trials and video collection occurred and schematic sketches of the experimental set up of baited pots. (A) Map data from GADM database of Global Administrative Areas (http://gadm.org/). Mercator projection WGS 84 was used. (B) Example of the configuration of part of a string of pots. (C) A two entrance Norwegian style-pot.

In this study, we investigate whether modifications to the Norwegian pot, designed to reduce the likelihood of entanglement in the entrance and side panels, affect capture rates of halibut. All species captured incidentally are also reported. Strings of experimental pots were deployed on halibut fixed gear commercial fishing grounds located in offshore waters of NAFO Division 0A (Fig. 1A). The objectives of our fishing trials were to determine if catch per unit effort (CPUE) of halibut differs among pots with varying design features (pot type) or by soak time. Additionally, we aimed to determine if bycatch differs among pot types. A large part of the success of experimental fishing gear relies on knowing how target and non-target species react to a new fishing gear so that potential obstacles to acceptance on the part of industry can be addressed (Anders et al., 2017; Fernö, 1993). For this reason, we also used video recordings to improve our knowledge of halibut behaviour in response to existing elements and modifications made to the entrance and frame of the Norwegian cod pot. The objective of our video work was to determine if halibut behaved differently when interacting with two different pot types.

## Materials and Methods

### Fishing trials

All pot types tested during this study were based on a commercially available two entrance Norwegian pot designed by the Institute of Marine Research (IMR) in Bergen, Norway to target Atlantic cod (Furevik et al., 2008; Ovegård et al., 2011). The Norwegian pot is fully collapsible with a horizontal panel (i.e., parlour entrance) that separates the pot into two chambers; a lower entrance chamber and an upper fish retention zone referred to here as the parlour (Fig. 1C). The parlour entrance was constructed with buoyant black polypropylene netting with a 58 mm mesh size and a longitudinal slit in the center of the netting that allows fish to swim into the parlour, while making it difficult for fish to find their way back into the entrance chamber. The entrance holes measured 28 cm ×16 cm (width × height). Pot dimensions were 1.5 m ×1.0 m ×1.2 m (length × width × height) with 12 mm galvanized round steel in the lower frame and 10 mm aluminum in the mid and upper frames. The exterior netting in the side and end panels of the pot were constructed with a thin black square nylon netting with a 58 mm mesh size and one mm diameter. The six shallow water Rosendahl floats described by Furevik et al. (2008) were replaced with two 20 cm diameter deep-water trawl floats with a working depth rating of 1,700 m and buoyancy rating of 2.3 kg. In addition, all salvages and mesh attachments to the frame of the pot were reinforced with two mm black mending twine.

This study tested three different diamond nylon netting mesh sizes in the entrance funnel of the Norwegian pot; (1) standard 50 mm clear monofilament (2) 19 mm clear monofilament and (3) three mm green twine. Halibut occur in cold boreal waters of the North Atlantic Ocean, with peak abundance in a depth range of 400–1,000 m (Bowering & Nedreaas, 2001). Fishers expressed concern that the light weight construction of the Norwegian pot would not withstand the rigors of fishing in deep water environments (i.e., >900 m) where they targeted halibut (Grant, 2015). For this reason, the three entrance funnel mesh sizes were also tested in a more durable, partially collapsible pot. The lower entrance chamber of the partially collapsible pot was constructed with 35 mm bar length ×3.5 mm diameter square PVC coated rigid wire mesh. Pots constructed with wire mesh are referred hereafter as wire pots, while pots constructed entirely with the original black nylon mesh will continue to be referred to as Norwegian pots. Fabrication of the wire pot simply involved attaching the horizontal separator panel and upper chamber from a Norwegian pot onto a lower chamber constructed of wire mesh. The wire mesh lower chamber was strengthened by attaching the 12 mm diameter galvanized round steel frame from a Norwegian pot to the base. All dimensions (i.e., length × width × height) were the same for each chamber of the wire pot and original Norwegian pots used in this study. Allowing the pot to be partially collapsible by maintaining nylon mesh in the upper chamber was seen as a means of conserving space on board commercial vessels, thereby allowing vessels to carry more pots. Abbreviations for the six pot types with either Norwegian (N) or wire (W) frames, and varying entrance meshes are as follows:

N_3mm_ = Norwegian pot with three mm green nylon entrance

N_19mm_ == Norwegian pot with 19 mm clear monofilament nylon entrance

N_50mm_ = Norwegian pot with 50 mm clear monofilament nylon entrance

W_3mm_ = Wire pot with three mm green nylon entrance

W_19mm_ = Wire pot with 19 mm clear monofilament nylon entrance

W_50mm_ = Wire pot with 50 mm clear monofilament nylon entrance

Two strings of pots were assembled, with each string containing five of each pot type, for a total of 30 pots. Pots were spaced at 55 m intervals (Fig. 1B) and baited with 1 kg of frozen squid (*Illex sp.)* using small mesh (two mm) bait bags hung in the center of the lower entrance chamber. The strings were deployed on offshore halibut fixed gear fishing grounds in NAFO Division 0A from 18–29 October 2016 (Fig. 1A).

This study took place on board a commercial fixed gear fishing vessel (*MV Kiviuq I*) and all pot strings were deployed and tended by experienced longline fishers. Potting experiments took place at the same time as the commercial gillnet fishery for halibut. Space to deploy the potting gear was made available between two gillnet fishing vessels that had been fishing on the grounds for three months. Each vessel was fishing 10 strings of baited gillnets that were 4.6 km in length and left to soak for 5 days at a time. Fleets of experimental pots were intended to soak for one night, but soak times ranged from 1 to 3 nights due to inclement weather. Overall, the mean soak time for experimental pots was 36 h and mean depth at the potting sites ranged from 1,008–1,278 m.

All fish caught in pots were identified to species level using Kulka, Miri & Thompson (2007) and Scott & Scott (1988). Total weight (± 1 kg) was obtained for each species and individual body lengths (± one cm) were recorded. Total-length was measured for fish without forked tails and fork-length was measured for fish with forked tails. Catch per unit effort was expressed as the total weight (CPUE_W_) and total number (CPUE_N_) of halibut captured in a pot. The weight contribution of each non-targeted species (i.e., percent bycatch) to the total catch weight was calculated for each pot.

All pots were visually inspected prior to redeployment. Damage such as torn netting or bending of the frame was repaired and heavily damaged pots were replaced. When damage was severe enough to allow escapement of fish, the data from that pot were excluded from analysis.

All data analysis was done using the software R (R Development Core Team, 2009), therefore all “packages” mentioned hereafter refer to data packages loaded in R. Each pot type was sampled though multiple deployments for each fleet of gear, therefore, for the CPUE data, Generalized Linear Mixed Models (GLMM) were used. GLMMs are able to handle unbalanced data that include a mix of random and fixed independent variables (Harrison et al., 2018). Independent variables for all CPUE models were pot type (fixed, categorical), soak time (fixed, categorical), and string (random, categorical) which was nested within deployment (random, categorical). The variable deployment refers to each time a fleet of gear is deployed from the research vessel. Soak time was treated as a categorical covariate (1 night, 2 nights, and 3 nights), which corresponded to soak codes 1, 2 or 3.

When comparing CPUE_w_, a Gaussian error structure was used because the dependent variable was a measurement (continuous). For CPUE_N_, the dependent variable was count data and a negative binomial error structure was found to be more fitting. The lme4 R package in version 1.1-17 (Bates et al., 2015) was used for fitting the models for CPUE_N_ and CPUE_W_. Analysis of percent bycatch per pot type was ran using the same fixed independent variables, but a beta error structure was required to fit the model since the dependent variable was now a proportion (percent weight of bycatch). This model was fit using the package betareg (Cribari-Neto & Zeileis, 2010).

To compare mean body lengths of halibut captured among pot types, a GLMM was used. The dependent variable was length (continuous), and independent variables were pot type (fixed, categorical) and string (random, categorical).

### Video observations

Most video sets were recorded at the same time and in the same area as fishing trials in NAFO Division 0A at a depth range of 828–1,254 m. Due to logistical reasons, additional underwater video recordings were collected at depths of 914–1,234 m on offshore halibut fixed gear fishing grounds in NAFO Division 0B from 7–13 October 2016 (Fig. 1A). Due to the challenges of deploying the camera equipment in deep water, we were only able to obtain sufficient video of halibut interacting with two pot types, N_50mm_ and N_19mm_.

Pots were attached to an aluminum observation frame to permit observations of the pot from above (Favaro, 2016). Two red Aquorea LED lights, built by SubC imaging for deep-water observations, were used for lighting. Red light is commonly used for deep-water observations since it is known to be less visible to fish and crustaceans than white light (Nguyen et al., 2017; Rooper et al., 2015). A 1Cam Alpha HD video camera and battery packs were securely attached to the frame, with the camera pointed downward toward the pot, giving an overhead view of the activity within 1 m of the pot. To avoid obstructing the overhead view of fish within a pot, the floats were removed before it was mounted to the camera frame. To keep the pot open, twine was used to tether the pot to the camera frame. Underwater video was recorded up to 20 h per day and the system was set to film in 30 min intervals, downloading video to an internal USB hard drive between sets to avoid data loss. The information gathered included, but was not limited to:

1. Approach—when a fish entered the camera field of view.
2. Direction of the approach relative to current direction ascertained from movement of particles in the water column (see Code Book in Supplementary Materials).
3. Encounter—a fish contacted the frame or netting.
4. Entry attempt—a fish entered an entrance funnel.
5. Entanglements and entanglement location—a fish became entangled in the netting in the entrance funnels, parlour entrance, or side panels.
6. Entry—a fish entered the entrance chamber.
7. Escape—a fish exited through an entrance funnel after entering.
8. Active (swimming rapidly about, bumping into netting and appeared to be seeking escape route) or neutral (resting on the floor of the entrance chamber or parlour) behaviour within the first 30 s of entering the lower entrance chamber or upper parlour.

Observational data was used to look at the relationship between the number of entanglements and the location in the pot where entanglements occurred. The model was fit using a GLMM with a negative binomial error structure in the lme4 package version 1.1-17 (Bates et al., 2015). The independent variables were the fixed variables location (entrance funnel, entrance chamber, parlour entrance, or parlour), pot type (N_19mm_ or N_50mm_) and the random factor deployment, in which entanglement location was nested. Further, chi-square tests were used to determine whether active or neutral behaviour were related to entrance type (19 mm or 50 mm monofilament) or location (entrance chamber or parlour).

Scores on halibut behaviour were used to calculate:

Encounter rate = *n*_*fov*_*/n*_*enc*_,

Entry rate = *n*_*cap*_/ *n*_*entr*_,

Escape rate = *n*_*esc*_/ *n*_*cap*_,

Parlour entry rate = *n*_*upp*_/ *n*_*cap*_, and

Capture rate = (*n*_*cap*_ *–n*_*esc*_)/ *n*_*entr*_

**Table 1:** Model summary outputs for best fitting catch models with conditional and marginal pseudo-R2 values.

Dependent variable	Marginal pseudo-R2	Conditional pseudo-R2	Random variables	Fixed variable	Estimate	Std. Error	*z*/*t* value	*p* value
CPUE _N_	0.443	0.736	Deployment	Intercept	1.728	0.106	16.288	<0.001
				Soak_Code2	0.656	0.084	7.778	<0.001
				Soak_Code3	0.310	0.163	1.895	0.058
				PotN19 mm	0.223	0.083	2.686	0.007
				PotN50 mm	0.068	0.088	0.773	0.439
				PotW3 mm	−0.116	0.089	−1.308	0.190
				PotW19 mm	0.167	0.084	1.980	0.047
				PotW50 mm	0.030	0.084	0.359	0.719
CPUE _W_	0.199	0.464	Deployment	Intercept	10.714	1.662	6.448	<0.001
			String	Soak_Code2	8.446	2.069	4.082	<0.001
				Soak_Code3	5.163	2.532	2.039	0.021
				PotN19 mm	3.647	1.271	2.869	0.002
				PotN50 mm	0.267	1.313	0.203	0.419
				PotW3 mm	−1.916	1.290	−1.485	0.069
				PotW19 mm	2.168	1.284	1.689	0.046
				PotW50 mm	−0.034	1.247	−0.027	0.489
Percent Bycatch	na	na	None	Intercept	−2.514	0.182	−13.795	<0.001
				Soak_Code2	0.492	0.174	2.836	0.005
				Soak_Code3	0.344	0.135	2.549	0.011
				PotN19 mm	0.194	0.206	0.942	0.346
				PotN50 mm	−0.246	0.211	−1.167	0.243
				PotW3 mm	−0.147	0.208	−0.708	0.479
				PotW19 mm	0.206	0.208	0.987	0.324
				PotW50 mm	−0.098	0.201	−0.485	0.628

where: *n*_*enc*_ = number that entered the video camera field of view; *n*_*fov*_ = number that came in contact with the frame or netting; n _entr_ = number that attempted to enter an entrance funnel; *n*_*cap*_ = number that successfully entered; *n*_*esc*_ = number that escaped; and *n*_*upp*_ = number that entered the parlour. T-tests were used to compare observed rates per deployment between the two pot types.

Since a fish could entangle multiple times, we explored the relationships among the number of entanglements observed at the pot entrance to the subsequent entanglements that occurred once fish were inside the pot. In addition, we considered whether a relationship between entanglements and escapement existed. For the relationship of entrance entanglement and subsequent entanglements within a pot, the dependent variable was number of observed entanglements after entering a pot, fixed effect was entanglement at the pot entrance (count), and the random effect was pot ID. The variable pot ID refers to the specific ID number associated to each individual pot. This model was fit using GLMMs with the lme4 package version 1.1-17 (Bates et al., 2015). For the effect of number of entanglements in a pot on escapement, the dependent variable was escapement and the fixed effect was the number of entanglements within the pot (count). This model was fit using a Generalized Linear Model (GLM) with a Poisson error structure.

All models that underwent model selection are listed in Tables 1 and 2. AIC values for all possible combinations of covariates and interaction terms were generated and the model with the lowest AIC value was used. To give indication on the amount of variance explained by fitted and random effects, conditional and marginal pseudo-R2 values were calculated for each mixed effect model (Nakagawa & Schielzeth, 2013) using the package MuMIn version 0.12.2 (Barton, 2009). If the conditional and marginal pseudo-R2 values for a model were equal, it was determined that the inclusion of random effects was not making significant improvements to the model, therefore a GLM was presented instead. Details on which covariates were used in the final models can be found in Tables 1 and 2. Assumptions were verified by plotting residuals versus fitted values, versus each covariate in the model and versus each covariate not in the model. Normality was assessed visually using quantile–quantile plots. Model validation indicated no problems. Tukey post hoc testing was used to determine where specific differences lied within significant independent variables.

**Table 2:** Model summary outputs for best fitting models for video observations with conditional and marginal pseudo-R2 values.

Dependent variable	Marginal pseudo-R2	Conditional pseudo-R2	Random variables	Fixed variables	Estimate	Std. Error	*z*/*t* value	*p* value
Entangle location	0.430	0.878	Deployment	Intercept	0.759	0.436	1.740	0.082
				LocationEntrance	0.797	0.255	3.127	0.002
				LocationOutside	−1.833	0.500	−3.664	0.000
				LocationParlour	0.351	0.268	1.313	0.189
				LocationParlourentrance	0.768	0.254	3.026	0.002
				PotN19 mm	−1.069	0.757	−1.412	0.158
				PotW50 mm	−0.156	0.203	−0.770	0.441
Subsequent	0.140	0.760	Deployment	Intercept	0.169	0.389	0.433	0.665
entanglements			Pot ID	Entrancefunnelentangle	0.242	0.070	3.440	<0.001
Escapement	na	na	None	Intercept	−1.808	0.531	−3.404	0.001
				Entanglement	−0.088	0.159	−0.553	0.580

This project was reviewed and approved by Memorial University’s Institutional Animal Care Committee (Project # 15-04-SG).

## Results

### Fishing trials

**Figure 2:**
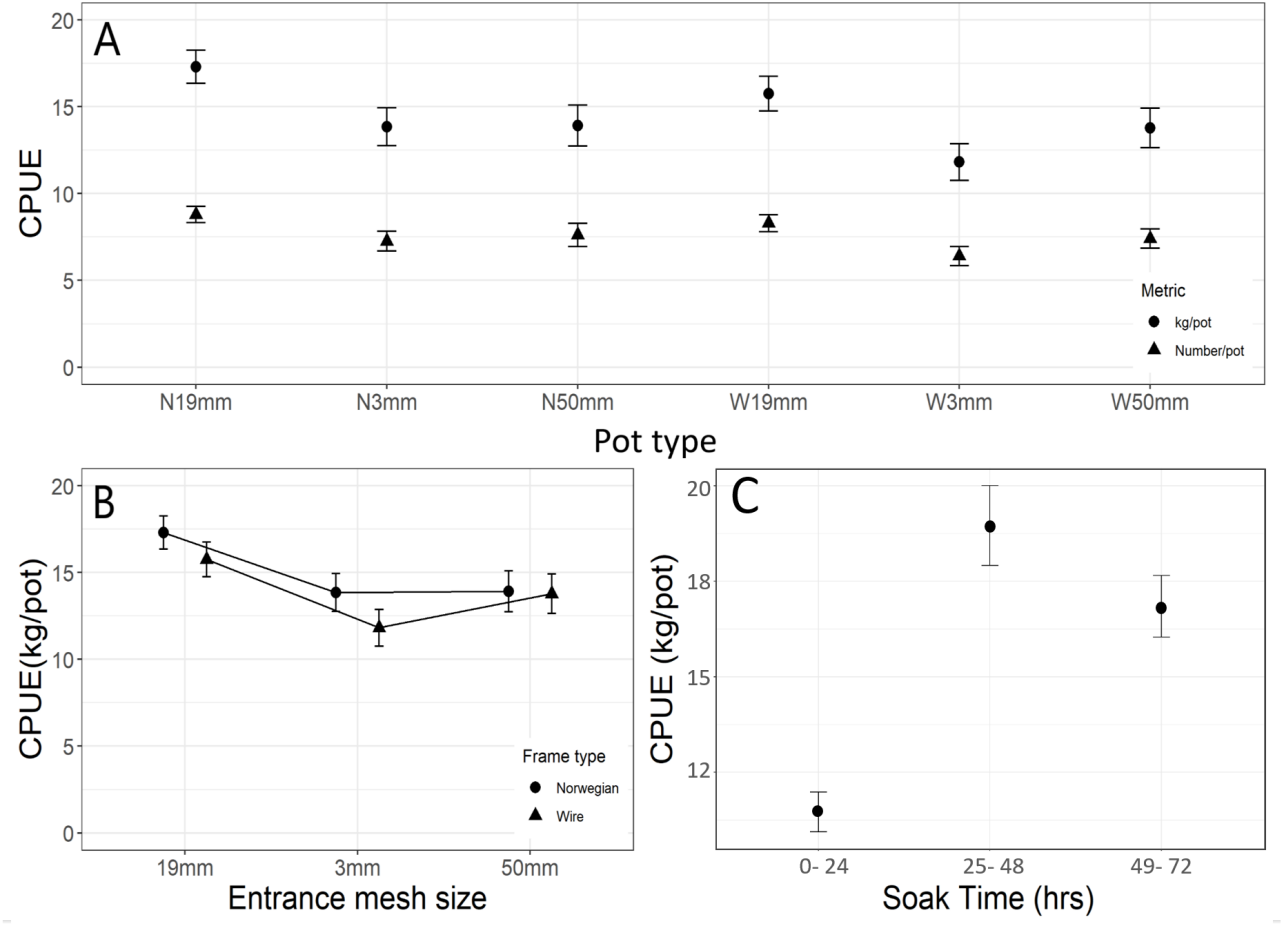
Catch per unit effort results for Greenland halibut in baited pots. (A) By pot type (means ± SE), (B) by entrance mesh size (means ± SE) and frame types, (C) by soak time (means ± SE).

A total of 2,439 halibut were caught in the 12 potting sets in NAFO Division 0A. Fifty-eight of these fish were omitted from the catch data because they were captured in damaged pots. Catches were highly variable within and among pot types (Fig. 2A) and mean catch per unit effort based on weight (CPUE_W_) and counts (CPUE_N_) differed significantly (*F*_5,302_ = 4.51, *p* = 0.005 and *F*_5,302_ = 3.95, *p* = 0.002 respectively) (Fig. 2A). The large differences in conditional and marginal pseudo-R2 values for the CPUE models suggest that random effects of deployment and string can account for a lot of the variability in our CPUE results (Table 1). For CPUE_N_, a Tukey post hoc analysis revealed significantly more halibut were captured in pots fitted with 19 mm entrances (N_19mm_ and W_19mm_) than in W_3mm_ pots (Fig. 2A). A similar trend was observed for CPUE_W_, with N_19mm_ pots significantly outperforming pots with green twine entrances (N_3mm_ and W_3mm)_ as well as wire pots with 50 mm entrances (W_50mm_) (Fig. 2A).

When CPUE_W_ was pooled across the wire and Norwegian pots for each entrance mesh size, catches differed significantly between entrance mesh sizes (*F*_2,307_ = 3.53, *p* = 0.031). Tukey post-hoc analysis revealed a significantly higher mean CPUEw in pots with the 19 mm entrance over that of the three mm entrance (*p* = 0.019) but not over the 50 mm (*p* = 0.215) (Fig. 2B). When CPUE _W_ was compared between the wire and Norwegian pots, catches did not differ significantly (*F*_1,308_ = 0.97, *p* = 0.326) (Fig. 2B).

**Figure 3:**
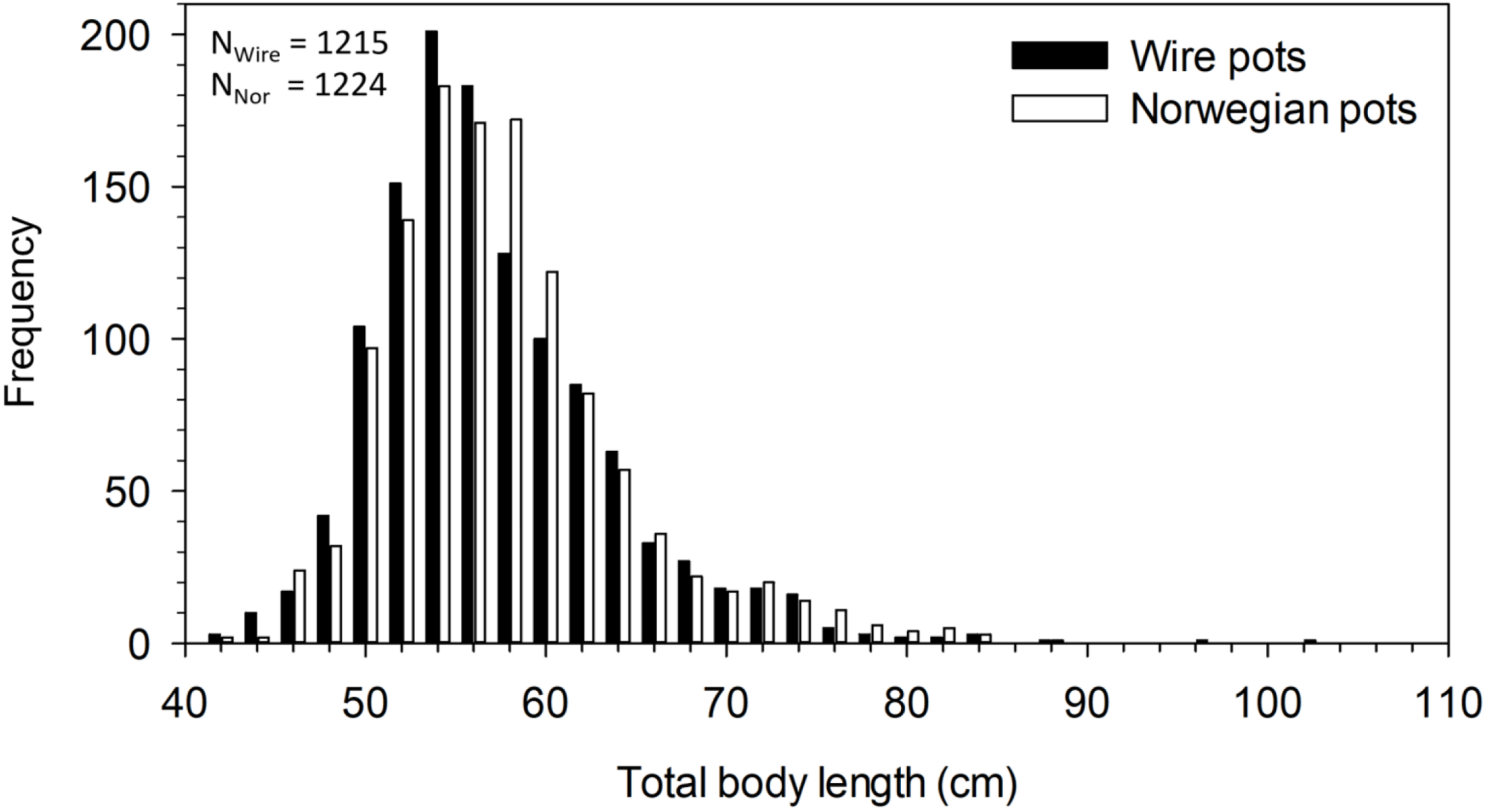
Length frequency distribution of halibut captured in wire and Norwegian pots in NAFO Division 0A. Total number of halibut measured for wire (N_Wire_) and Norwegian (N_Nor_) pots is also shown.

The mean total body length of all halibut captured in pots was 56.8 cm. Body length differed significantly between pot types (*F*_5,2433_ = 2.408, *p* = 0.035). Post-hoc analysis revealed the difference was between catches in the N_19mm_ and W_3mm_ pots, with halibut in the former being 1.6 cm longer than in the latter (*p* = 0.027). A comparison of the mean body length among all wire pots combined (i.e., 56.5 ±6.5 cm, *n* = 1215) and all Norwegian pots combined (i.e., 57.1 ±6.8 cm, *n* = 1224) found fish caught in Norwegian pots to be significantly longer in mean body length (*t*_2437_ = 2.21, *p* = 0.027) (Fig. 3). Although statistically significant, these differences in size are minor and of little consequence.

In total, 31 of the 2,439 halibut captured during this study were dead when the pots were hauled aboard the vessel (1.3%). Most mortalities (77%) were entangled by their teeth in netting (Table 3). An additional 19 halibut were entangled in the netting when pots were hauled and, although they were not dead, they exhibited a limited physical response when grasped by the caudle peduncle. Overall, 43 (1.8%) of the halibut captured were entangled in the netting when pots were hauled. Thirty-five of these fish were entangled in nylon netting in the side and end panels and the parlour entrance; eight were entangled in the 50 mm entrance funnel. There were no halibut entangled in the 19 mm or three mm entrance funnels at the time of haul back. All remaining halibut captured in pots were alive and active when the pots were hauled and exhibited an active response when grasped by the caudal peduncle. We did however record 12 halibut that exhibited scars, mucus loss, scale loss, and bruising patterns, which may be indicative of escaping or falling out of commercial gillnets that were set in close proximity to the potting sites.

**Table 3:** Counts of entanglements and deaths of Greenland halibut observed at haul back during fishing trials in NAFO division 0A.

	Total	Alive	Dead
Captured	2,439	2,408	31
Entangled	43	19	24
58 mm Nylon netting (side panels and parlour entrance)	35	14	21
50 mm entrance	8	5	3
19 mm entrance	0	0	0
3 mm entrance	0	0	0

No significant interaction was found between soak time and pot types for CPUE _N_ (*F*_10,294_ = 0.59, *p* = 0.441) or CPUE _W_ (*F*_10,294_ = 1.28, *p* = 0.243). However, soak time did have a significant effect on overall CPUE_W_ (*F*_2,307_ = 19.73, *p* <0.001). Pots hauled in the 25–48 h soak period (2 nights/ soak code 2) caught on average 7.5 kg more halibut than the pots hauled in the 0-24 h (1 day/ soak code 1) soak time intervals (Table 1, Fig. 2C). Our results indicate strings set for two nights with the best performing pot (N _19mm_) would yield a mean CPUE _N_ of 11.9 and mean CPUE _W_ of 21.1 kg of halibut per pot.

### Bycatch

Eight species were captured incidentally during the experiments (Table 4). Bycatch of all non-targeted species combined expressed as a percentage of total catch weight per pot did not differ significantly among the six treatments (*F*_5,304_ = 0.29, *p* = 0.918). Northern wolffish (*Anarhichas denticulatus*), three-bearded rockling (*Gaidropsarus ensis*), and silver rockling (*Gaidropsarus argentatus*) dominated the bycatch by weight accounting for 39%, 31%, and 13% respectively of all non-targeted species. The rocklings were most prevalent in numbers, exhibiting the highest frequency of occurrence among pot types followed by the Northern wolffish. No Greenland sharks were captured in the pots used in this study. However, three large (2–3 m) Greenland sharks were observed interacting with the pots in the video analysis, yet none tried to bite or break into pots, nor did they become entangled in or damage the pots.

**Table 4:** Summary of the mean percent of the total catch weight (%Total) for non-targeted species captured in experimental pot treatments in NAFO Division 0A. The percentage of pots that captured each species is also shown (%Pot).

	W_3mm_		W_50mm_		W_19mm_		N_3mm_		N_50mm_		N_19mm_	
Species	%Tot	%Pot	%Tot	%Pot	%Tot	%Pot	%Tot	%Pot	%Tot	%Pot	%Tot	%Pot
Three-bearded rockling (*Gaidropsarus ensis*)	2.40	30.0	2.83	33.3	3.18	35.3	4.71	44.4	2.44	31.9	3.75	54.7
Silver rockling (*Gaidropsarus argentatus*)	0.98	14.0	2.23	28.1	1.09	27.5	1.41	31.5	0.77	23.4	1.63	41.5
Northern wolffish (*Anarhichas denticulatus*)	5.72	12.2	2.65	8.8	5.23	13.7	4.95	15.4	4.05	14.9	2.14	7.5
Spotted wolffish (*Anarhichas minor*)	2.33	4.1	0.86	3.5	0.27	2.0	0	0	0.29	2.1	0.95	5.7
Thorny skate (*Amblyraja radiata*)	0.91	6.0	0.85	5.3	0.69	5.9	0.66	5.6	0.96	6.4	0.41	5.7
Polar eelpout (*Lycodes polaris*)	0.09	4.0	0.14	10.5	0.19	13.7	0.03	3.7	0.03	2.1	0.10	11.3
Roughhead grenadier (*Macrourus berglax*)	0	0	0.33	5.3	0.16	3.9	0	0	0.45	4.3	0.22	5.7
Polar sculpin (*Cottunculus microps*)	0.04	2.0	0.01	1.8	0	0	0.05	3.7	0.04	6.3	0	0
Total (all species)	12.47		9.89		10.81		11.81		9.03		9.19	

All wolffish captured in baited pots were alive, active (exhibiting physical resistance and biting reflex when grasped by the caudal peduncle), and in good physical condition with no external wounds. Similarly, three-bearded rockling, silver rockling, Thorny skate (*Amblyraja radiata*), polar eelpout (*Lycodes polaris*), and polar sculpin (*Cottunculus microps*) captured in baited pots were alive and active when handled and did not exhibit external wounds. When handled, spinytail skates curled their tails over the body and they maintained this posture as they descended into the water. Roughhead grenadier (*Macrourus berglax*) was the only species to experience barotrauma, appear moribund, and not descend when returned to the ocean.

### Video observations

In total, eight camera sets (83 h of video) of the N_50mm_ pot and four camera sets (55 h of video) of the N_19mm_ pot were analyzed. A summary of the visual information gathered for each pot type is illustrated in Table 5. Overall, fewer halibut were observed to approach the N_19mm_ pots and mean approach rates were 1.1 fish/h compared to 5.6/fish per hour in the N_50mm_ pots.

**Table 5:** Underwater video observation summary illustrating the total number of hours of video observed, total number of approaches, encounters, entry attempts, successful entries, escapes, parlour entries, and entangles by teeth and location for two pot treatments.

	N_19mm_	N_50mm_	Total
Video duration (hrs)	55	83	138
Approaches	68	383	451
Encounters	28	138	166
Entry Attempt	25	114	139
Successful entry	14	34	48
Escapes	3	15	18
Enter parlour	11	12	23
Entangle by teeth			
Outer side panel of entrance chamber	2	3	5
Entrance funnel	7	60	67
Inner side panel of entrance chamber	7	25	32
Parlour entrance	16	53	69
Inner side panels of parlour	13	33	46
Total	45	174	219

**Figure 4:**
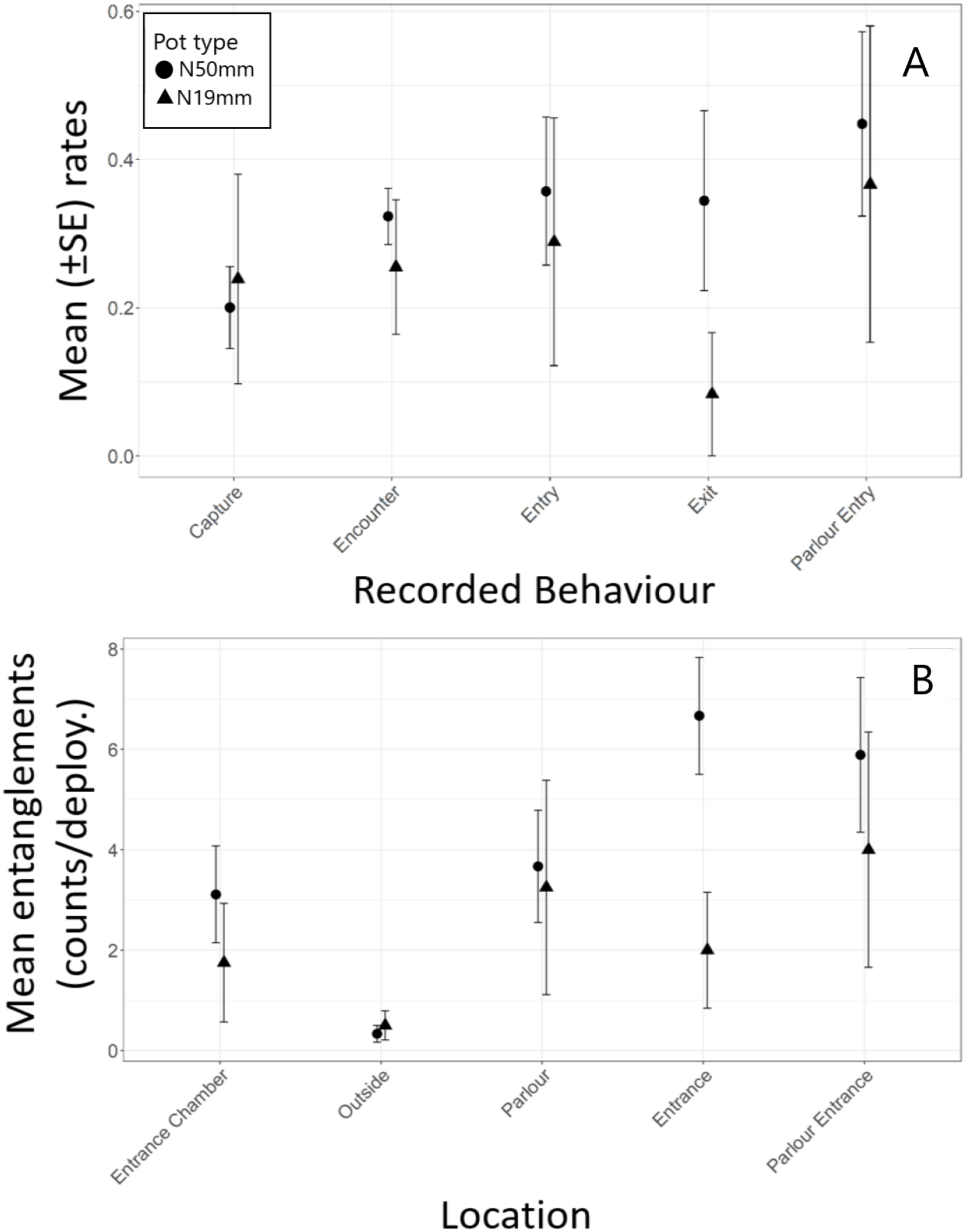
Summary of Greenland halibut behaviour while interacting with baited N_19*mm*_ and N_50*mm*_ pots. (A) Rates of each Behaviour type (mean ± SE). (B) Observed counts of entanglements (mean ± SE) by location in the pot.

Most (80%) of the halibut approached pots against the current (Table 6). The downstream entrance of the experimental pots was aligned with the bottom current in 52% of the approaches. Overall, 70% of entry attempts and 67% of successful entries occurred when the entrance was aligned with the current and when fish approached a pot against the current. Analysis indicated there was no significant difference in encounter rate (*t*_11_ = 0.84, *p* = 0.418), successful entry rate (*t*_8_ = -0.83, *p* = 0.431), escape rate (*t*_8_ = 0.80, *p* = 0.449), parlour entry rate (*t*_8_ = -0.87, *p* = 0.412), or capture rate (*t*_8_ = −2.09, *p* = 0.070) between the N_50mm_ and N_19mm_ pots (Table 7, Fig. 4A). However, successful entry rates were in fact 45% higher in the N_19mm_ pots and escape rates were 56% lower, which resulted in a 109% increase in capture rates when compared to the N_50mm_ pots (Table 5).

**Table 6:** Greenland halibut approach direction relative to the bottom current.

Approach direction	Number of approaches	Percent
Against current	361	80.0%
With current	45	10.0%
Cross-current	45	10.0%
Total	451	

**Table 7:** Mean (±S.E.) rates of encounter, entry, escape, parlour entry and capture for Greenland halibut in two pot treatments.

	N_50mm_	N_19mm_	Totals
Encounter rate	0.32 (± 0.11)	0.25 (± 0.18)	0.30 (± 0.13)
Entrance rate	0.40 (± 0.29)	0.58 (± 0.31)	0.40 (± 0.26)
Escape rate	0.39 (± 0.36)	0.17 (± 0.24)	0.31 (± 0.34)
Parlour entrance rate	0.50 (± 0.36)	0.73 (± 0.09)	0.50 (± 0.33)
Capture rate	0.23 (± 0.16)	0.48 (± 0.11)	0.25 (± 0.18)

Halibut commonly entangled in the nylon netting in the pots and most of the entanglements were by their teeth (Table 5). However, two fish were also observed to become entangled by their tail in the parlour entrance. Halibut entangled significantly more often in the entrance funnel and the entrance to the parlour then in other areas of the pot (Table 2, Fig. 4B). Tukey post hoc analysis indicated halibut entangled significantly more often in the entrance funnel of the N_50mm_pots than in the N_19mm_ pots (*F*_1,11_ = 5.82, *p* = 0.034) (Fig. 4B).

All of the fish that became entangled in the entrance funnels of both pot types exhibited active behaviour, rapidly twisting and turning and moving forward and backward until they were able to free themselves within 6 to 187 s (mean = 43 s) with 41% exiting the entrance funnel and rapidly swimming out of the camera’s field of view. The other 59% entered the entrance chamber with 77% continuing to exhibit active behaviour by rapidly swimming about. Linear mixed model analysis revealed the number of entanglements observed after a fish entered a pot was significantly related to entrance entanglement (Table 2) but the number of entanglements that occurred after fish entered a pot did not appear to influence escapement (Table 2). However, we were not able to track all fish individually throughout their residency within a pot. Observations of behaviour prior to a fish escaping were limited to immediately before the escapement. In some cases, halibut behaviour (in terms of entanglement rates) differed considerably between deployments and Pot ID’s, as evident by the difference in the marginal and conditional pseudo-R2 values for these models (Table 2). Overall, the number of entanglements of halibut by their teeth ranged from 1 to 12 (mean = 4.6) per deployment.

Overall, 37% (18 individuals) of the halibut that entered the pots were observed to escape through the entrance funnel (Table 5). Sixty-one percent of these fish exited within one minute of entering a pot, 17% exited within an hour, and the remainder exited over the next 1–4 h. Four of the fish that exited within one minute were observed to enter one entrance and swim directly out the opposite entrance. Of the fish that exited a pot, 67% (12/18) exhibited active behaviour immediately prior to exiting and 42% (5/12) of these fish were observed to entangle in the netting immediately prior to escapement. Chi-square tests revealed no significant difference in active behaviour within the first 30 s upon entering a pot among fish in the N-_50mm_ and N_19mm_ pots (*X*^2^_(2)_,  *N* = 70 () = 2.34, *p* = 0.126). Sixty-one percent of fish were active in the N_50mm_ pots compared to 41.7% in the N_19mm_ pots. Fish in the entrance chamber (where 66.7% of all fish were active) were found to be significantly more active than those in the parlour (where 27.3% were active) (*X*^2^_(2)_,  *N* = 70 () = 9.43, *p* = 0.002).

On four separate occasions we were able to observe the behaviour of halibut as a pot was hauled to the surface. When fished in a string, pots are attached to the ground line by a lanyard that is tied to the steel frame on the bottom of a pot which results in pots ascending through the water column at about 45° . This resulted in the fish falling back into the lowest section of the parlour or entrance chamber of a pot. None of the halibut made any effort to escape when hauling commenced or as a pot ascended 914 –1,254 m to the surface.

## Discussion

### Fishing trials

Baited pots, deployed in close proximity to heavily baited gillnets, efficiently caught up to 38.4 kg of Greenland halibut within a single pot. Catch results demonstrate that a substantial quantity of halibut can be captured in the N_19mm_ baited pots during 48 h soaks, with a mean CPUE_N_ and CPUE_W_ of 11.9 fish and 21.1 kg, respectively.

It should be considered that conducting potting experiments in such close proximity to heavily baited gillnets likely influenced catch results. Further trials with no gillnets in use should be done to accurately compare catch rates among future pot designs. Atlantic cod can detect and locate a longline chemical bait source from distances of 600–700 m (Løkkeborg, Bjordal & Fernö, 1989). Additionally, Kallayil et al. (2003) demonstrated that acoustically tagged Atlantic cod could be attracted to a string consisting of two baited gillnets from 400–800 m away. Given this, we suspect that during the current study, the increased level of baiting carried-out by halibut gillnet fishers negatively influenced the availability of halibut to the potting gear. These conclusions are supported by the lower mean approach rates of halibut (i.e., 1.1 fish/h) in video observations when a baited pot was set in close proximity to baited gillnets in NAFO Div. 0A compared to mean approach rates (i.e., 5.5 fish/h) when a baited pot was set on fixed gear fishing grounds that had been abandoned for the season in NAFO Div. 0B.

Bayse & Grant (2020) demonstrated that baiting gillnets used to target halibut in offshore waters of Davis Strait (NAFO Div. 0B) increased catch rates by 150–254% (depending on bait technique) over non-baited gillnets, and catch rates of some non-targeted species were also significantly higher in baited gillnets. Indeed, halibut landings (i.e., catch excluding discards) in individual strings of 50 baited gillnets set adjacent to the potting sites during the current study were about 9,000–10,000 kg per string (Captain M. Letto, 2016, pers. comm.). This corresponds to 108–120 kg when standardized to a 55 m section of a string of gillnets which was also the distance between pots in a string. These standardized gillnet catch rates were 5.1–5.7 × the maximum mean catch rate observed in 48 h soaks of the N_19mm_ pots during this study (21 kg/pot). The higher catch rates in baited gillnets can be explained by a number of factors including: increased concentration of chemical attractants in the baited gillnets (i.e., a bait bag every 9–18 m in gillnets vs. every 55 m in a string of pots), horizontal range of attraction relative to increased chemical concentration and vertical distance bait was off the seabed (i.e., 2.5 m in gillnets vs. 0.3 m in baited pots), extended soak time of gillnets (i.e., five or more days vs. 1–3 days in baited pots), and continuous release of feeding attractants by self-baiting gillnets resulting from dead and decomposing fish. Further, depending on swimming direction, halibut would have to navigate through several strings of baited gillnets before they encountered our baited pots. Lastly, with pots, the likelihood of escapement probably increases with increasing soak time, thereby reducing overall catch rates. This effect does not occur with gillnets, which entangle rather than trap fish.

Overall, the fishing trial results of this study show catch rates that may not reflect real-world performance because of the presence of baited gillnets. We conclude ambiguity was created with the results in regards to the performance of the pots and relationships between catch rate and pot type.

### Bycatch

Fishing results from this study suggest that halibut pots have relatively low bycatch rates in comparison to gillnets (Bayse & Grant, 2020) , and overall no pot type caught significantly more non-target species than the others. It is unclear whether setting pots in close proximity to heavily baited gillnets affected the incidental capture of non-targeted species. For example, Bayse & Grant (2020) demonstrated that significantly more rocklings (*Gaidropsaurus sp.*) and Northern wolffish were captured in baited versus non-baited gillnets. The same species were captured incidentally in Norwegian pots by Grant (2015) in the absence of baited gillnets. Both studies (current and (Grant, 2015)) show that apart from species that exhibit barotrauma due to the presence of a swim bladder (i.e., grenadier), all remaining species were in good physical condition, alive, and active when the pots were tended, suggesting high post-release survival(Benoît & Hurlbut, 2010). Spotted and northern wolffish are SARA (Species at Risk Act) listed species, therefore future studies should seek to verify survival of wolffish and other vulnerable pot caught fish once returned to the ocean (e.g., Grant & Hiscock, 2014).

The original Norwegian pot tested by Grant (2015) was found to both decrease bycatch of Greenland shark and outperform longlines, the recommended fixed gear for targeting halibut in inshore waters of Nunavut (DFO, 2014). In the current study, very little interactions of Greenland sharks with pots were observed and none were captured as bycatch. This further supports the idea that pots are an efficient gear type to mitigate bycatch of Greenland sharks in the halibut fishery. In the future, it is recommended to test the effects of halibut potting within the NSA (inshore waters of Nunavut) where high numbers of Greenland shark may be present (Pike, 1994; Young 2010; Devine, Wheeland, & Fisher 2018; Grant, Sullivan & Hedges, 2018).

### Video observations

This study was part of an ongoing effort to develop a deep-water pot that is suitable for capturing large quantities of halibut while minimizing the incidental capture and depredation caused by non-targeted species including Greenland shark. We hypothesized that the use of alternate netting materials in various components of the original Norwegian pot would prevent entanglement of halibut and increase the number of halibut that successfully entered a pot. Recorded video observations did not suggest a significant difference in capture rates between the two pot types. However, significantly fewer halibut entangled in the entrance funnels of the N_19mm_ pots than in the N_50mm_ pots. It should be noted that the deployment sample sizes (N_19mm_, *n* = 4; N_50mm_, *n* = 8), likely lowered the power of these analyses, limiting the statistical comparison conducted.

Our results suggest that netting in entrance funnels and in parlour entrances need to be substituted with smaller (i.e., 19 mm or less) mesh to prevent entanglement, increase the rate of capture, and reduce active behaviour that can lead to injury or stress. Stress in fish due to entanglements most likely reduces fish quality, as capture stressors and physical injuries due to contact with gear or other fish are more likely to occur when fish are entangled (Chopin & Arimoto, 1995; Huss et al., 1995; Humborstad et al., 2016; Savina et al., 2016). Another potential negative aspect of halibut entangling in the mesh of pots is that fish in distress could be an attractant to predators such as wolffish and Greenland shark (Løkkeborg, Bjordal & Fernö, 1989). Alternatively, a halibut in distress in the entrance could deter other halibut from entering the pot (Bagdonas, Humborstad & Løkkeborg, 2012). Video observation data confirmed halibut that entangled in entrances were more likely to entangle in additional pot locations once they entered. Further, decreasing the amount of entanglements in the palour entrance of the pot would facilitate movement into the parlour where fish were shown to be less active than in the entrance chamber. Our catch results indicated that at haul back, halibut mortality is high in entangled fish. This demonstrates the importance of considering proper mesh size to reduce entanglements in the entrance as well as subsequent entanglements.

Halibut also entangled frequently in the 58 mm polypropylene netting used in the side and end panels of the pots. However, at the risk of increasing the capture of undersized halibut, it would not be recommended to reduce the size of the 58 mm nylon netting around the side and end panels. The 58 mm polypropylene netting had a thin, one mm mesh diameter and was not positively buoyant. Polyethylene netting however, is positively buoyant and would assist in opening the collapsible pots on the seabed and facilitate escapement of small fish through mesh openings (Meintzer, Walsh & Favaro, 2018). Meintzer, Walsh & Favaro (2018) used a 100 mm polyethylene mesh with a diameter of three mm, in the side and end panels of the Norwegian pot to target Atlantic cod, which successfully reduced the capture of undersized cod. We suspect that a buoyant netting with less slack could reduce entanglement of halibut in the side and end panels, therefore we suggest future halibut potting studies consider testing this alternative netting.

It was originally thought that the use of a rigid wire mesh in the lower chamber would both strengthen the section of the pot that was in contact with the seabed and eliminate entanglement of halibut. We were not able to observe whether there was a reduction in entanglements, however the damage wire pots sustained and suspected seabed impact due to weight could be reason enough to omit the design from further studies. Repairing damaged wire pots requires the use of specialized equipment, making them difficult and costly to mend at sea. Alternatively, damage to Norwegian pots, to either the frame or netting, is much easier to address. It is also important to consider that fully collapsible Norwegian pots take up less space on board, making them easier to accommodate on smaller vessels used in the NSA.

The majority of halibut approached a baited pot against the current providing evidence of chemically mediated rheotaxis (Løkkeborg, 1998). This study corroborates previous studies highlighting the importance of having the entrance funnel aligned with the prevailing bottom current (Anders et al., 2017; Løkkeborg, 1998; Meintzer, Walsh & Favaro, 2017) as the majority of the entry attempts and successful entries occurred when halibut approached a pot against the current and when an entrance was aligned with the current. When targeting Atlantic cod in the Baltic Sea, providing adequate floatation to lift a single entrance Norwegian-style pot 0.5 m off the seabed has been shown to improve entry alignment (Jørgensen et al., 2017; Königson et al., 2015a). However, we suspect there would be considerable operational limitations of setting strings of several floating pots in deep waters inhabited by halibut.

An alternative to floating pots above the sea bed would be to develop halibut pots with a greater number of entrances, increasing the likelihood of at least one entrance aligning with bottom currents. Meintzer, Walsh & Favaro (2018) developed a four-entrance pot and in experiments conducted in coastal waters of Newfoundland they demonstrated that this pot could capture about 30% more Atlantic cod than the traditional two entrance Norwegian cod pot. However, Meintzer, Walsh & Favaro (2018) speculated that exit rates may be equal to or even greater than entrance rates and in the current study we observed cases where halibut passed directly through the pot by swimming in one entrance and directly out of the opposite entrance. Given the escape rates observed in the current study, use of a four-entrance pot may require additional modifications.

Changing the configuration of the entrance funnels so that openings into the entrance chamber are vertically and horizontally offset from each other would likely prevent fish from swimming directly out of an adjacent entrance. Alternatively, some trap fisheries use one-way entrance retention devices to prevent escapement of target species (Carlile, Dinnocenzo & Watson, 1997; Salthaug, 2002). These devices are placed at the end of the entrance funnel and have finger like projections or triggers that hang vertically and pivot in one direction (i.e., inward) or taper inward from the top and bottom. Murphy (2014) used vertically hanging one-way entrance devices constructed of steel to target halibut in baited pots. Although very few halibut were captured, the capture of several American plaice (*Hippoglossoides platessoides*) suggests vertically hanging triggers do not negatively influence the entry of flatfish (Murphy, 2014). The introduction of a four-entrance pot with light weight vertically hanging triggers in the entrance would ensure one entrance is aligned with bottom current at all times, while preventing escapement of captured fish through entrance funnels.

## Conclusions

The results of this study suggest that CPUE of halibut does vary by both soak time and pot design. Alternatively, bycatch rates were not affected by pot designs and remained low. Halibut behavior did vary between the two pot designs that were observed during video trials, with significantly fewer entangling in the N_19mm_ pots than in the N_50mm_ pots. Preeminently, this study demonstrates the value of *in situ* observations for guiding decisions regarding the best features to incorporate into a baited pot to minimize entanglement and stress, as well as maximize catch rates of targeted species. Ultimately, this study shows that the use of catch rate data alone can increase the risk of drawing erroneous conclusions.

Mean halibut capture rates of 21.1 kg/pot in the best performing pots (N_19mm_) at optimal soak times (48 h) may not meet the expectations of current offshore or inshore fisheries. However, if suggested modifications derived from this study are used, pots may be found to outcompete open water longline fisheries in inshore areas of Nunavut, where gillnets are prohibited (i.e., Cumberland Sound). Additional video work with future pots should be conducted in coastal waters to determine the most suitable design before it is introduced to offshore waters or compared to baited gillnets.

##  Supplemental Information

10.7717/peerj.10536/supp-1 Supplemental Information 1 Catch data for all Greenland halibut and bycatch species captured in the six pot types during experimental fishing trials in October 2016

10.7717/peerj.10536/supp-2 Supplemental Information 2 Species weight contribution of bycatch species captured in the six pot types during experimental fishing trials in October 2016

10.7717/peerj.10536/supp-3 Supplemental Information 3 Length data for all Greenland halibut and bycatch species captured in the six pot types during experimental fishing trials in October 2016

10.7717/peerj.10536/supp-4 Supplemental Information 4 Video data recorded while observing fish behaviour in response to potting gear meant to target Greenland halibut

10.7717/peerj.10536/supp-5 Supplemental Information 5 Code book for some variables recorded in the raw video data

## Additional Information and Declarations

### Field Study Permissions

The following information was supplied relating to field study approvals (i.e., approving body and any reference numbers): This project was reviewed and approved by Memorial University’s Institutional Animal Care Committee (Project # 15-04-SG).

### Data Availability

The following information was supplied regarding data availability: Raw data are available in the Supplemental Files.
