# Supplementary material for: A feasibility study to determine the use of baited pots in Greenland halibut (Reinhardtius hippoglossoides) fisheries, supported by the use of underwater video observations
Source: PeerJ. 2021 Jan 4;9:e10536. doi: 10.7717/peerj.10536 (PMC7789868; doi:10.7717/peerj.10536)

Code Book for Video data

| Variables | Code | Description |
| --- | --- | --- |
| Current direction | U | approached from upper side of pot |
| and | UL | approached from upper left corner of pot |
| Approach direction | UR | approached from upper right corner of pot |
|  | D | approached from lower side of pot |
|  | DL | approached from lower left corner |
|  | DR | approached from lower right corner |
|  | L | approached from left (in line with left entrance) |
|  | R | approached from right (in line with right entrance) |
| Approach direction relative to current | us | Upstream - Fish approach within 90°, travelling against the current |
|  | ds | Downstream - Fish approach within 90°, travelling with the current |
|  | cc | Cross current - Any approach not considered us or ds |


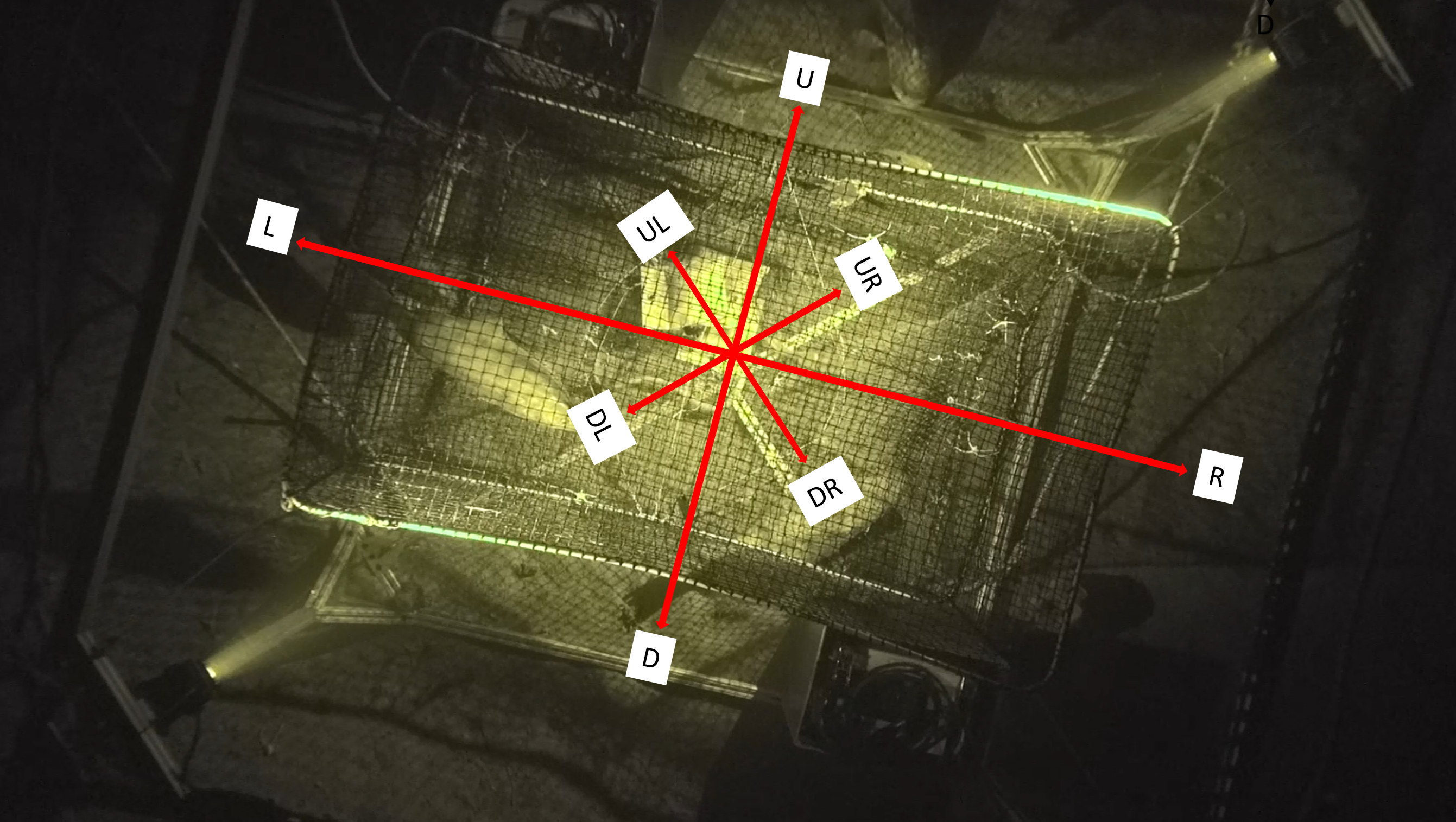

Supplement: Supplemental Information 5 [file peerj-09-10536-s005.docx]
